# Receptor Tyrosine Kinases in Osteosarcoma Treatment: Which Is the Key Target?

**DOI:** 10.3389/fonc.2020.01642

**Published:** 2020-08-28

**Authors:** Zhichao Tian, Xiaohui Niu, Weitao Yao

**Affiliations:** ^1^Department of Orthopedics, The Affiliated Cancer Hospital of Zhengzhou University, Henan Cancer Hospital, Zhengzhou, China; ^2^Department of Orthopedic Oncology, Beijing Jishuitan Hospital, Beijing, China

**Keywords:** TKIs, RTKs, target therapy, VEGFRs, RET, osteosarcoma

## Abstract

Recent clinical trials have shown several multi-target tyrosine kinase inhibitors (TKIs) to be effective in the treatment of osteosarcoma. However, these TKIs have a number of targets, and it is yet unclear which of these targets has a key role in osteosarcoma treatment. In this review, we first summarize the TKIs that were studied in clinical trials registered on ClinicalTrials.gov. Further, we compare and discuss the targets of these TKIs. We found that TKIs with promising therapeutic effect for osteosarcoma include apatinib, cabozantinib, lenvatinib, regorafenib, and sorafenib. The key targets for osteosarcoma treatment may include VEGFRs and RET. The receptor tyrosine kinases (RTKs) MET, IGF-1R, AXL, PDGFRs, KIT, and FGFRs might be relevant but unimportant targets for osteosarcoma treatment. Inhibition of one type of RTK for the treatment of osteosarcoma is not effective. It is necessary to inhibit several relevant RTKs simultaneously to achieve a breakthrough in osteosarcoma treatment. This review provides comprehensive information on TKI targets relevant in osteosarcoma treatment, and it will be useful for further research in this field.

## Introduction

Osteosarcoma is the most common primary bone malignancy (1). For decades, the treatment model for osteosarcoma has not advanced much. The treatment includes neoadjuvant chemotherapy, surgery, and postoperative chemotherapy, and patients have a 5-years survival rate of about 60% (2). Patients who experience metastatic disease have limited options; they have expected 4-months progression-free survival (PFS) rate of 12% only (3). In recent years, with the great success of tyrosine kinase inhibitor (TKI) use in the treatment of cancers, the treatment for osteosarcoma has entered a new phase.

**Figure 1:**
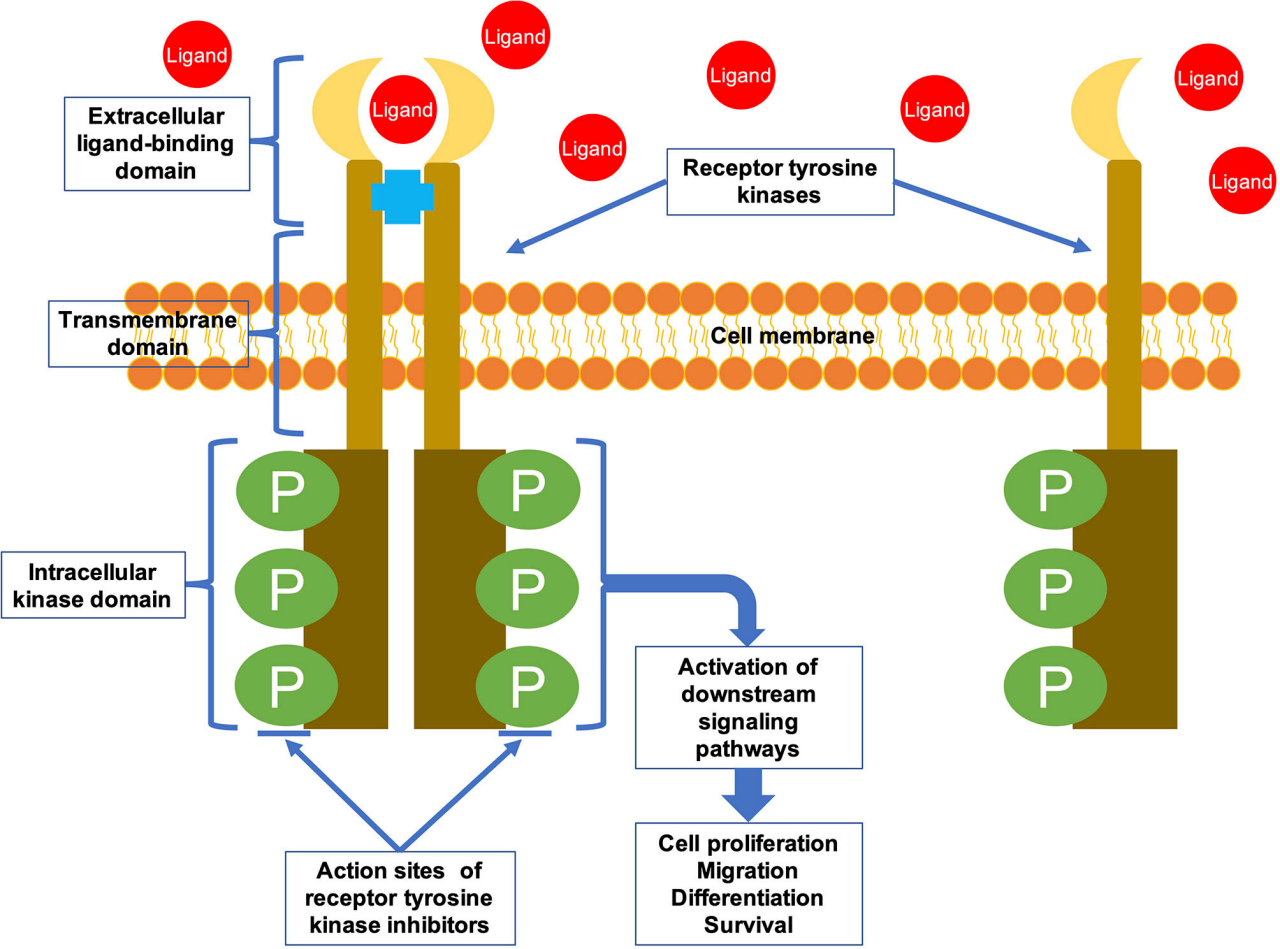
The general structural characteristics and activation mechanism of an RTK. RTKs are transmembrane glycoproteins that consist of an extracellular ligand-binding domain, a transmembrane domain, and an intracellular kinase domain. They are activated by ligand binding and then transduce the extracellular signal to the cytoplasm by phosphorylating tyrosine residues on the receptors themselves (autophosphorylation) and on downstream signaling proteins.

TKIs are targeted drugs that can specifically inhibit protein tyrosine kinases (PTKs). PTKs are important signaling molecules which having highly regulated activity and are critical components of signaling pathways that control cellular differentiation and proliferation (4). There are 90 PTKs that encoded by human genome (5). These PTKs can be classified into 32 species of non-receptor tyrosine kinases (NRTKs) and 58 species of receptor tyrosine kinases (RTKs) (6). RTKs are involved in the process of extracellular signals into cell, whereas NRTKs regulate intracellular communication (7). RTKs are transmembrane glycoproteins that consist of an extracellular domain, a transmembrane domain, and an intracellular kinase domain (Figure 1) (8). They are activated upon binding to their ligands, and the extracellular signal is transduced to the cytoplasm by phosphorylation of the tyrosine residues on the receptors themselves and on downstream signaling proteins (8). RTKs activate multiple signaling pathways leading to cell migration, differentiation, proliferation, and metabolic changes (9). NRTKs are downstream signaling molecules of RTKs and various other receptors. They do not have transmembrane domains, and are located in nucleus or cytoplasm. NRTKs can be phosphorylated by different RTKs or trans-autophosphorylated upon dimerization (10).

RTK irregularities can lead to a range of diseases. More than half of the oncogene and proto-oncogene products have RTK activities, and their abnormal expression leads to the disorder of cell regulation, which eventually leads to tumorigenesis (11). In addition, RTK irregularities are related to tumor neovascularization, invasion, metastasis, and chemotherapy resistance (4). Therefore, RTKs have become a focus of research on antitumor drugs. Most TKIs are small molecule inhibitors designed to interfere with binding of the RTK intracellular domain with ATP or other substrates, thereby inhibiting the catalytic activity of RTKs (Figure 1) (12, 13). Many of the TKIs inhibit several RTKs as the intracellular domain is relatively conserved among RTKs (14). The targeted drugs that showed efficacy in osteosarcoma treatment are these multi-target TKIs (15).

In this review, we first summarize the TKIs that have shown results in registered clinical trials of osteosarcoma treatment. Further, we compare and discuss the targets of these TKIs. This comprehensive review will be a valuable resource for further research on the use of TKIs in osteosarcoma treatment.

## TKIs Reported in Registered Clinicaltrials for Osteosarcoma Treatment

TKIs reported in registered clinical trials on patients with osteosarcoma that have been conducted so far are listed in Table 1. Clinical trials were included herein only if: (1) they were prospective clinical trials, (2) they were registered with ClinicalTrials.gov, (3) their subjects were osteosarcoma patients, (4) the administered pharmacotherapy exclusively included TKIs, with no other drugs, and (5) those with detailed results were retrievable on https://pubmed.ncbi.nlm.nih.gov. There are retrospective studies on other TKIs (pazopanib and sunitinib) in the treatment of osteosarcoma (35, 36). We have not listed them here as retrospective nature is a limitation of such studies.

**Table 1:** Targets and clinical outcomes of TKIs which have shown results in registered clinical trials of osteosarcoma treatment.

**TKIs**	**RTKs and IC**_****50****_ **(nM, mean)**								**Clinical outcome**	**References**
	**VEGFR1**	**VEGFR2**	**VEGFR3**	**KIT**	**RET**	**PDGFRα**	**PDGFRβ**	**FGFR1**		
Apatinib	70	1	-	429	13	>1,000	-	>1,0000	PR rate 43% (16/37), 4-months PFS rate 56.76%, m-PFS 4.5 m.	(16, 17)
Axitinib	0.1	0.2	0.29	1.7	>1,000	5	1.6	231	5-months *SD* rate 100% (2/2).	(18, 19)
Cabozantinib	12	0.035	6	4.6	5.2	-	234	5,294	PR rate 12% (5/42), 6-months PFS rate 33%, m-PFS 6.2 m.	(20–22)
Cediranib	5	<1	3	2	-	36	5	26	One of four patient had PR after two cycles.	(23, 24)
Imatinib	19,500	10,700	5,700	97	-	72	-	31,200	Five of 27 patients had *SD* at 4 months.	(25–27)
Lenvatinib	4.7	3	2.3	85	6.4	29	-	61	PR rate 8% (2/26), 4-months PFS rate 33%, m-PFS 3.4 m.	(28, 29)
Regorafenib	13	4.2	46	7	1.5	-	22	202	PR rate 8% (2/26), 12-weeks PFS rate 62%, m-PFS 16.4 w.	(30, 31)
Sorafenib	-	4	20	68	0.4	18	57	580	PR rate 9% (3/35), 4-months PFS rate 46%, m-PFS 4 m.	(32–34)

*PFS, progression-free survival; TKI, tyrosine kinase inhibitor; RTKs, receptor tyrosine kinases; PR, partial response; VEGFR, vascular endothelial growth factor receptor; KIT, stem cell factor receptor; RET, rearranged during transfection; FGFR1, fibroblast growth factor receptor; PDGFR, platelet-derived growth factor receptor; SD, stable disease*.

### Apatinib

**Figure 2:**
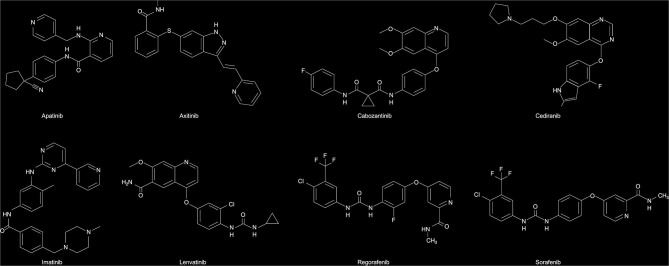
The chemical structure of protein tyrosine kinases.

Apatinib (Figure 2) was approved for the treatment of metastatic or advanced gastric cancer in China in 2014 (37). Because of its low price, it is widely used off-label in China for treating various malignant tumors, including osteosarcoma (17, 37, 38). Apatinib has fewer confirmed targets compared to other multi-target TKIs; these targets include vascular endothelial growth factor receptor-1 (VEGFR1, IC_50_ = 70 nM), VEGFR2 (1 nM), rearranged during transfection (RET, 13 nM), stem cell factor receptor (KIT, 429 nM), and v-src avian sarcoma (Schmidt-Ruppin A-2) viral oncogene homolog (SRC, 530 nM) (Table 1) (16).

In the treatment of osteosarcoma, preclinical studies have shown that apatinib promotes apoptosis and autophagy through VEGFR2/STAT3/BCL-2 signaling pathways in osteosarcoma cells (39), and it inhibits invasion, migration, and PD-L1 expression in osteosarcoma cells (40). Apatinib also attenuates doxorubicin-induced cancer stemness and cancer cell migration of osteosarcoma cells by inhibiting Sox2 (41). Only one phase II trial has been conducted on patients with advanced osteosarcoma (17). In this open-label clinical trial, 37 patients with advanced or metastatic osteosarcoma after failure of standard chemotherapy, received 500 or 750 mg of apatinib once daily in accordance with their body surface area until unacceptable toxicity or disease progression was observed. Consequently, the 4-months PFS was 57% among the apatinib-treated patients, with a median PFS of 4.5 months, and a marked 43% partial response (PR) rate. Doses were reduced or interrupted among 25 of 37 (67%) patients, owing to drug toxicity. The most common grade 3–4 adverse events (AEs) were pneumothorax (16%), wound dehiscence (10%), proteinuria (8%), diarrhea (8%), and palmar-plantar erythrodysesthesia syndrome (8%). No treatment-related deaths were observed. The PR rate in this trial was significantly higher than that reported by retrospective studies (42, 43). It may be due to the high dose of apatinib, which was 750 mg per day in the registered trial, compared with 500 mg per day in retrospective studies. In conclusion, apatinib is an important and effective TKI in osteosarcoma treatment.

### Axitinib

Axitinib (Figure 2) is an orally administered TKI of VEGFR1 (0.1 nM), VEGFR2 (0.2 nM), VEGFR3 (0.1–0.3 nM), platelet-derived growth factor receptor-α (PDGFRα, 5.0 nM), PDGFRβ (1.6 nM), Colony stimulating factor 1 receptor (CSF-1R, 73 nM), KIT (1.7 nM), fibroblast growth factor receptor (FGFR1, 231 nM), and RET (>1,000 nM) (Table 1) (18). Axitinib was developed for the treatment of many solid malignancies. It was approved by the Food and Drug Administration (FDA) of the United States to treat advanced renal cell carcinoma (RCC) after failure of other systematic therapies in 2012 (44).

There is no preclinical evidence of axitinib in the treatment of osteosarcoma, and only one clinical trial of axitinib in the treatment of osteosarcoma (19). This phase 1 and pilot consortium trial was about the safety and efficacy of axitinib in adolescents with recurrent or refractory solid tumors. In this trial, 19 patients received axitinib twice daily in continuous 28-days cycles. The most common (>20%) grade 1–2 AEs during treatment included anorexia, anemia, diarrhea, fatigue, hypertension, and nausea. Non-hematological toxicity of grade ≥3 AEs included elevated serum lipase levels and hypertension. Drug exposure and dosage were not associated with hypertension. Five patients presented with stable disease (SD) as the optimal response, including two osteosarcoma patients. One patient with alveolar soft part sarcoma presented a partial response. This result suggests that axitinib has limited efficacy in osteosarcoma compared to other TKIs listed in Table 1. Perhaps that is why there have been few studies on axitinib for osteosarcoma.

### Cabozantinib

Cabozantinib (Figure 2) is a fairly multiple target TKI with activity not only for VEGFR2 (0.035 nM) but also for the N-methyl-N0-nitroso-guanidine human osteosarcoma transforming gene RTK (MET, 1.3 nM), KIT (4.6 nM), RET (5.2 nM), VEGFR3 (6 nM), anexelekto RTK (AXL, 7 nM), VEGFR1 (12 nM), and PDGFRβ (234 nM) (21, 22). Registered clinical trials of cabozantinib for prostate (45), lung (46), renal cell (47), and thyroid cancers have been completed (48). It was approved by FDA for the treatment of advanced or metastatic medullary thyroid cancer, RCC and hepatocellular carcinoma (HCC) (49).

In osteosarcoma, preclinical studies have shown that cabozantinib is able to inhibit proliferation and migration of osteosarcoma cells through the ERK and AKT signaling pathways (50). The French sarcoma group had carried out a multicenter, phase II trial involving patients with advanced or metastatic osteosarcoma after failure of other systemic therapy (20). Ninety osteosarcoma patients received cabozantinib orally every day continuous 28-days cycles until unacceptable toxicity or disease progression was observed. The median follow-up duration was 31 months among osteosarcoma patients. Forty-two (93%) osteosarcoma patients were assessable for treatment efficacy through histological and radiological analyses. Five (12%) osteosarcoma patients presented a PR and the median PFS was 6.2 months. The most frequent grade 3 or 4 AEs were hypophosphatemia, increased aspartate aminotransferase levels, palmar-plantar syndrome, pneumothorax, and neutropenia. No treatment-related deaths occurred. The longer median-PFS results represent the best achievement to date in the treatment of osteosarcoma by TKIs.

### Cediranib

Cediranib (Figure 2) is an oral multi-target TKI with targets including VEGFR1 (5 nM), VEGFR2 (<1 nM), VEGFR3 (3 nM), KIT (2 nM), PDGFRβ (5 nM), FGFR-1 (26 nM), and PDGFRα (5.0 nM) (Table 1) (23). Cediranib has shown promising activity in a series of malignancies (51), including alveolar soft-part sarcoma, in preclinical models and in clinical trials (52); however, these results are not sufficient to get approval for its routine use until now.

Just like axitinib, there is no preclinical evidence of cediranib being efficient to treat osteosarcoma, and only one phase I trial has been conducted on patients with osteosarcoma (24). In this trial, 16 patients were recruited. Grade 3 or 4 AEs included nausea, vomiting, fatigue, hypertension, and prolonged corrected QT interval. Grade 1 or 2 AEs included palmar-plantar erythrodysesthesia, left ventricular dysfunction, weight loss, elevated thyroid stimulating hormone levels, and headache. One of four patients presented with minor PR as the optimal response. The results were similar to those of axitinib. This may be because the two drugs have similar targets and target sensitivity (Table 1).

### Imatinib

Imatinib (Figure 2) is significantly different from the other TKIs presented in Table 1. The main difference is that imatinib does not affect VEGFR1 (19,500 nM), VEGFR2 (10,700 nM), VEGFR3 (5,700 nM), and FGFR1 (31,200 nM). Its sensitive targets include KIT (97 nM), PDGFRα (72 nM), and CSF-1R (291 nM) (25). Imatinib was approved by FDA for the treatment of chronic myeloid leukemia and advanced or metastatic gastrointestinal stroma tumors (GIST) (53, 54).

In preclinical studies, imatinib inhibits proliferation of osteosarcoma cells and inhibits tumor growth in preclinical murine models of osteosarcoma (55). However, another study suggests that imatinib is effective in osteosarcoma treatment only when imatinib is combined with another therapeutic intervention, such as induction of cellular stress, that renders cell survival dependent on PDGF signaling (56). In a phase II multicenter trial on patients with osteosarcoma administered with imatinib (27), five of 27 osteosarcoma patients achieved SD at 4 months after treatment initiation, and various genes in the KIT/PDGFR pathway were either aberrantly expressed or mutated, suggesting that imatinib was not effective against advanced-stage osteosarcoma. This undesirable outcome has led to the exclusion of imatinib from clinical treatment options for osteosarcoma.

### Lenvatinib

Lenvatinib (Figure 2) is an oral multi-target TKI with targets including VEGFR1 (4.7 nM), VEGFR2 (3 nM), VEGFR3 (2.3 nM), PDGFRα (29 nM), KIT (85 nM), FGFR-1 (61 nM), and RET (6.4 nM) (Table 1) (28). Phase I trials of lenvatinib were conducted simultaneously worldwide, for the treatment of thyroid cancer, endometrial cancer, RCC, melanoma, colon cancer, and sarcoma (57). Lenvatinib is approved by FDA for the treatment of advanced HCC, radioiodine-refractory differentiated thyroid cancer, and advanced RCC (58).

There are no preclinical studies of lenvatinib in osteosarcoma. An ongoing clinical trial of lenvatinib in relapsed osteosarcoma reported initial results (29). In this trial, patients were aged 2–25 years, and had undergone <2 previous targeted therapies. Twenty-six patients received 14 mg/m^2^/day of lenvatinib. The most common AEs were diarrhea, proteinuria and hypothyroidism, and the most common grade 3 or 4 AEs were dyspnea and back pain. The 4-months PFS was 33%, the median PFS was 3.4 months, and the PR rate was 8%. This result indicates that lenvatinib has activity against osteosarcoma, and further studies will most probably prove this.

### Regorafenib

Regorafenib (Figure 2) is an orally administered multi-target TKI that targets VEGFR1 (13 nM), VEGFR2 (4.2 nM), VEGFR3 (46 nM), PDGFRβ (22 nM), KIT (7 nM), FGFR-1 (202 nM), and RET (1.5 nM) (Table 1) (30). Regorafenib is the first TKI that showed efficacy in advanced or metastatic colorectal cancer (59), and was approved in 2012 by FDA for this indication. In addition, regorafenib is approved by FDA for the treatment of GIST and HCC (60, 61). It has also been examined in several different tumor types, including relapsed glioblastoma (62), RCC and soft-tissue sarcoma (63, 64). And additional phase II trials in other solid malignant tumors are ongoing (65).

*In vitro* experiments have shown that regorafenib inhibits osteosarcoma cell growth by inducing apoptosis of cells (66). Two registered clinical trials have been reported on regorafenib in osteosarcoma. The first is a non-comparative, placebo-controlled phase II trial about the efficacy and safety of regorafenib in metastatic osteosarcoma patients (31). In this trial, 38 patients with metastatic osteosarcoma after failure of one or two previous lines of chemotherapy were enrolled. Patients were randomly assigned to receive either regorafenib or a matching placebo. Non-progression was observed in 65% (17 of 26) patients in the regorafenib group. The 12-weeks PFS was 62%, the median PFS was 16.4 weeks, and the PR rate was 8%. The most common grade 3 or 4 AEs included hypertension, hand–foot skin reactions, fatigue, hypophosphatemia, and chest pain. No treatment-related deaths occurred. Another clinical trial was similar to the above (3). This trial enrolled 42 patients with advanced or metastatic osteosarcoma, after failure of at least one prior line of therapy. Study enrolment was terminated early, upon review by the data safety monitoring committee. The median PFS was significantly higher in the regorafenib group than that in the placebo group. And the efficacy and safety were similar to the above trial. These two studies met their primary end point, demonstrating the efficacy and safety of regorafenib in patients with advanced or metastatic osteosarcoma after failure of prior therapy.

### Sorafenib

As a multi-target TKI, the targets of sorafenib (Figure 2) include VEGFR2 (4 nM), VEGFR3 (20 nM), PDGFRβ (22 nM), KIT (68 nM), FGFR-1 (580 nM), and RET (0.4 nM) (Table 1) (32, 33). In 2005, sorafenib was approved by FDA for the treatment of advanced RCC (67). Moreover, it was the first TKI approved for patients with radioiodine-refractory differentiated thyroid cancer (68).

Sorafenib is the most studied multi-target TKI in osteosarcoma. Preclinical studies have found that sorafenib blocks tumor growth, tumor angiogenesis, and tumor metastatic potential of osteosarcoma in preclinical murine models (69). Another study has shown that sorafenib targets the PTKs RET and VEGFR2 and suppresses cell proliferation (31). Two registered clinical trials have been reported on sorafenib in osteosarcoma. The first is a phase II trial of sorafenib in patients with advanced or metastatic osteosarcoma after failure of standard treatment (34). In this trial, 35 patients were enrolled, and the 4 months PFS rate was 46%, the median PFS was 4 months, and the PR rate was 9% (3/35). Sorafenib treatment was reduced or briefly interrupted in 46% patients owing to toxicity. Another clinical trial yielded similar results (70). These two clinical trials are milestone achievements for the treatment of advanced osteosarcoma, because they are the first multicenter prospective clinical trials to demonstrate the efficacy of TKIs in osteosarcoma.

## Which RTK Is the Key Target?

**Figure 3:**
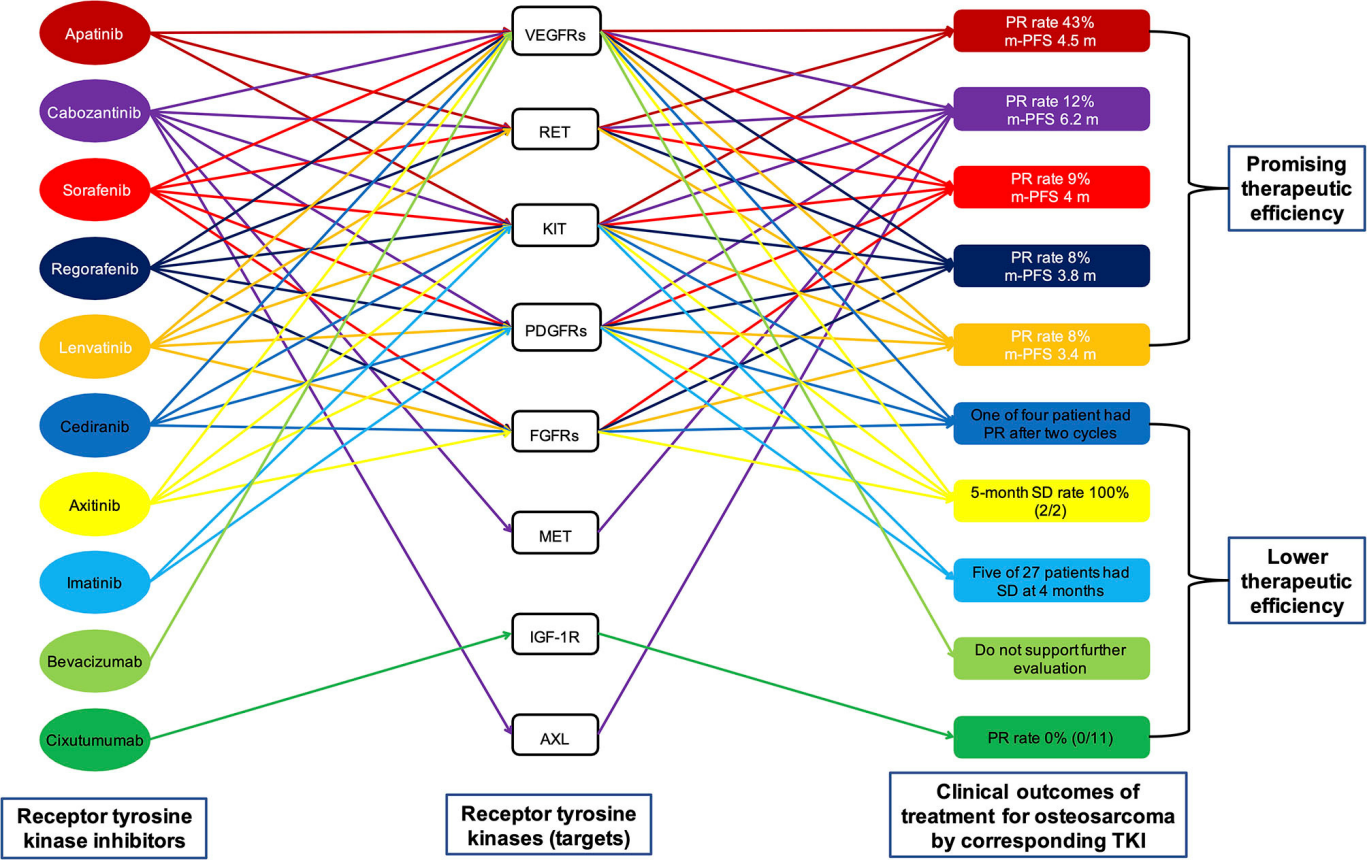
A visual interaction map of the different targets of the different drugs. Preclinical data indicate that all eight targets shown in the figure play an important role in the progression of osteosarcoma. However, clinical trials in osteosarcoma have demonstrated the low efficacy of single-target therapy by inhibiting VEGFs (by bevacizumab), KIT and PDGFRs (by imatinib), and IGF-1R (by cixutumumab). The results of these clinical trials suggest that the inhibition of one type of targets in the treatment of osteosarcoma is not feasible. PFS, progression-free survival; TKI, tyrosine kinase inhibitor; PR, partial response; VEGFR, vascular endothelial growth factor receptor; KIT, stem cell factor receptor; RET, rearranged during transfection; FGFR1, fibroblast growth factor receptor; PDGFR, platelet-derived growth factor receptor; SD, stable disease.

The targets of the aforementioned multi-target TKIs, namely RTKs, are listed in Table 1 and Figure 3. It can be seen that only KIT is the common target of all multi-target TKIs mentioned above. Other RTKs that are targets of majority TKIs include VEGFRs, RET, PDGFRs, and FGFR1. To determine which RTK is the key target for osteosarcoma, we continue to review the relevant literature.

### VEGFRs

VEGF is a highly specific vascular endothelial growth factor, which can promote vascular permeability, extracellular matrix degeneration, endothelial cell migration, proliferation, and angiogenesis (71). VEGF signaling pathways have three RTKs, namely VEGFR1, VEGFR2, and VEGFR3 (72). VEGFR1 has the strongest binding affinity to VEGF and more thorough phosphorylation, but it has less effect on the activation of intracellular signaling intermediates (73). VEGFR2 is the dominant receptor that mediates the biological effect and pro-angiogenic functions of VEGF, which is closely related to cell division and chemotaxis (74). And this pathway has been under research focus on priority for the development of antiangiogenic therapies (75). VEGFR3 exists only in lymphoid endothelial cells in adults. In a variety of cancer types, VEGFR3 is associated with tumor lymphangiogenesis, lymph node invasion and metastasis (76). Currently, anti-angiogenic therapies targeting VEGF/VEGFRs signaling pathway include anti-VEGF antibodies (Bevacizumab) (77), anti-VEGFR2 antibodies (ramucirumab) (78), and TKIs (14). Both bevacizumab and ramucirumab have been approved by FDA for use in therapy of a series of malignancies. At present, more than a dozen types of TKIs that target VEGFRs have been approved by FDA for the treatment of cancer therapy.

Regarding osteosarcoma, a systematic review showed that high VEGF expression in osteosarcoma patients were associated with lower PFS rate. In addition, osteosarcoma patients with high VEGF expression were associated with shorter overall survival (OS) (79). These findings suggested that targeting the VEGF signaling pathway may be an effective treatment for osteosarcoma. However, this is not the case. The clinical outcomes of targeting VEGF/VEGFR signaling pathways in osteosarcoma are not always favorable. The clinical efficacy of bevacizumab in osteosarcoma was limited (Figure 3) (80), and there is not even a single study on the clinical treatment of osteosarcoma with ramucirumab until now. As shown in Table 1 and Figure 3, TKIs—including apatinib, cabozantinib, lenvatinib, regorafenib, and sorafenib—having better therapeutic effect on osteosarcoma include VEGFRs as targets, whereas TKI (imatinib) whose targets did not include VEGFRs had poor therapeutic effect on osteosarcoma. This seems to prove that VEGFRs are essential for TKIs to be effective in osteosarcoma treatment. However, not all multi-target TKIs that target VEGFRs are effective in treating osteosarcoma; for example, axitinib and cediranib (Table 1 and Figure 3). In conclusion, therapy that targets VEGF/VEGFR is an important and effective anticancer method. However, the efficiency of inhibiting just VEGF/VEGFR in osteosarcoma treatment is limited.

### KIT

The KIT is a kind of RTK, and its ligand is stem cell factor (81). KIT also contains an extracellular domain, a transmembrane domain, and an intracellular domain (82). After binding with a ligand, KIT induces multiple signaling cascades, which are essential for biological processes including cell differentiation, proliferation, and migration (83). KIT plays important roles in the functioning of nervous system, kidney, gastrointestinal tract, bones, lungs, and pancreas (81). Abnormal expression of KIT has been documented in various neoplasms (82, 84). These results have encouraged researchers to work on the use of KIT as a potential target in the treatment of malignant tumors.

Although KIT has been widely studied in neoplastic and non-oncological diseases, there are few preclinical and clinical studies on KIT in osteosarcoma. In 2007, a study showed that tumor specimens from 20 out of 100 patients with osteosarcoma were KIT-positive and that OS were not different between patients with and without KIT expression (85). In 2011, another study found that KIT was expressed in 46.15% patients with osteosarcoma and that KIT-positive tumors had worse response to chemotherapy (86). The results of both studies point to the possibility that KIT is not a key player in proliferation and metastasis of osteosarcoma. Although all the TKIs effective in osteosarcoma listed in Table 1 and Figure 3 include KIT as a target, there are still three TKIs (axitinib, cediranib, and imatinib) that include KIT as a target but are not effective in osteosarcoma. Based on the limited evidence described above, we surmise that KIT is not a key RTK in osteosarcoma treatment and that targeting KIT alone in osteosarcoma treatment is inefficient.

### RET

The RTK RET is coded by the gene RET (87). As an RTK, RET also has characteristic composition of extracellular domain, transmembrane domain, and intracellular kinase domain (88). RET binds with the ligand–coreceptor complex of glial cell line-derived neurotrophic factor family ligands and glial cell line-derived neurotrophic factor family receptor alpha (89). The RET-bound complex then leads to phosphorylation of intracellular kinase domain, which activates downstream signaling pathways. RET signals are associated with many RET-mediated functions (90). RET mutations have been documented in a variety of organ systems (91–93). Abnormal expression of RET has been observed in many malignancies (94, 95). These results have led to the use of RET as a capable target in cancer therapy. Several multi-target TKIs targeting RET are approved by the FDA for cancer therapy and non-oncologic disease treatment (94). Selective RET inhibitors selpercatinib and pralsetinib are undergoing clinical trials respectively, with preliminary results demonstrating partial response and low incidence of serious AEs (96, 97).

The role of RET in osteosarcoma has been far less studied than in other malignancies. Using phosphoproteomic screening, RET was first identified *in vitro* as a receptor that can promote behavior of metastatic osteosarcoma cells and is thought to be a potential therapeutic target for osteosarcoma (98). It has also been confirmed that the overexpression of RET is associated with chemotherapeutic resistance induced by cisplatin and bortezomib, and with the increase of stem cell-like properties of osteosarcoma (99–101). There are many clinical trials on the role of RET in osteosarcoma. As shown in Table 1 and Figure 3, all multi-target TKIs (apatinib, cabozantinib, lenvatinib, regorafenib, and sorafenib) with high efficiency in osteosarcoma are inhibitors of RET. Further, the three multi-target TKIs (axitinib, cediranib, and imatinib) with low efficiency in osteosarcoma do not inhibit RET. In conclusion, RET is an important and potentially critical target for osteosarcoma treatment, and it may be equally important as VEGFRs. The role of RET in osteosarcoma has not been studied in detail and warrants further study.

### PDGFRs

PDGF is named for its origin in platelets. It exists in normal physiological state in platelets. When blood clots, PDGF is released by disintegrated platelets and activated (102). PDGF signaling pathway consists of four ligands and two receptors (PDGFR-α and PDGFR-β) (103). Ligand-induced receptor dimerization of PDGF receptors leads to autophosphorylation of the receptors (103, 104). A series of downstream signaling molecules bind to specific phosphotyrosines in the intracellular domain of PDGFRs and mediate intracellular signal transduction (105, 106). Activation of these pathways leads to cell proliferation and migration (103, 107). PDGFs/PDGFRs are expressed in many normal human cells and are involved in the normal development and physiological process of important organs (105). The PDGFs/PDGFRs pathway play an important role in the development and metastasis of cancers (104). Given the association between PDGF over-activity and malignant tumors, different kinds of PDGF inhibitors have been developed. These inhibitors include the fully human IgG1 monoclonal antibody against PDGFRα named olaratumab (108), and multi-target TKIs such as cediranib, imatinib, and sorafenib (Table 1).

Many studies have confirmed that PDGFs/PDGFRs signaling pathway play an important role in the proliferation and migration of osteosarcoma (109, 110). However, the expression of PDGFs/PDGFRs in osteosarcoma has a significant heterogeneity (111), and the association of PDGFs expression with osteosarcoma prognosis has not yet been established. Some studies have suggested that high expression of PDGFs is associated with poor prognosis of osteosarcoma patients (111, 112). On the contrary, other studies have shown that OS and PFS analysis was not different between osteosarcoma patients on the basis of PDGFs/PDGFRs expression level (113, 114). The use of PDGFR inhibitor alone in clinical trials is not efficacy, whereas its combination with other drugs seems more promising (27, 115–117). As shown in Table 1 and Figure 3, axitinib and cediranib are effective inhibitors of PDGFRs, yet their efficacy in osteosarcoma is as poor as imatinib. Apatinib, which is more effective in osteosarcoma, does not target PDGFRs. The PDGFs/PDGFRs signaling pathway may not to be the driver of osteosarcoma, especially in the presence of other signaling pathways such as VEGFs. Therefore, the application of PDGFs/PDGFRs inhibitor would be expected to be effective only if used in combination with another drug.

### FGFRs

FGFRs are a subfamily of RTKs, with four subtypes including FGFR1, FGFR2, FGFR3, and FGFR4 (118). As RTKs, FGFRs also has characteristic composition of extracellular domain, transmembrane domain, and intracellular kinase domain that transduces downstream signaling (119). The ligands of FGFRs comprise 22 growth factors, which are classified into seven subgroups (120). FGFs/FGFRs signaling pathway are activated through FGFs binding to FGFRs, which depends on heparan sulfate, a glycosaminoglycan (121). Activation of FGFRs results in receptor dimerization, transphosphorylation of receptor kinase domains, and activation of downstream signaling pathways (122, 123). FGFs/FGFRs signaling pathway play an important role in many physiological processes such as embryogenesis, tissue repair, wound healing, and inflammation (119). Aberrant activation of the pathway due to FGFRs amplification, mutations, or gene fusions has been observed in many malignancies (124). Currently, several multi-target or selective TKIs that target FGFRs have been approved by FDA for the treatment of malignancies (125, 126).

Regarding osteosarcoma, a study revealed low expression of FGFR-2 and FGFR-3 across standard and patient-derived osteosarcoma cells (127). Another study, which included 352 osteosarcoma samples, detected FGFR1 amplification in 18% of the chemotherapy resistant osteosarcoma patients (128). This suggests that FGFR1 amplification is associated with poor response of osteosarcoma to chemotherapy. Another study showed that deregulated FGFRs signaling plays an important role in osteosarcoma formation and the development of lung metastases (129). There are no clinical trials of selective FGFR inhibitors in osteosarcoma. There are many clinical trials of multi-target FGFR TKIs in osteosarcoma. As shown in Table 1 and Figure 3, cediranib, a multi-target FGFR1 TKI, is not effective in the osteosarcoma treatment. However, apatinib and cabozantinib, which do not target FGFR1, can effectively treat osteosarcoma. To sum up, it is clear that FGFR signaling pathways might be relevant but is unimportant in osteosarcoma treatment.

## Other RTKs in Osteosarcoma

By summarizing the clinical trials of various multi-target TKIs in osteosarcoma and their targets, we have identified the above five RTKs that may be associated in the treatment of osteosarcoma. However, the key RTKs for osteosarcoma seem to be more than that.

### MET

MET kinase is a heterodimer protein (130). As an RTK, MET contains a characteristically extracellular domain, transmembrane domain, and intracellular kinase domain (131). The binding of its ligand, hepatocyte growth factor, results in MET dimerization, transphosphorylation of receptor kinase domains, and activation of downstream signaling pathways (132). MET signaling pathway play an important role in many physiological processes (132, 133). Aberrant activation of the pathway due to MET amplification, mutations, or gene fusions has been observed in many malignancies and degenerative diseases (133, 134). Several small-molecule inhibitors and monoclonal antibodies of MET are in the development (134).

Regarding osteosarcoma, a study has shown that overexpression of the MET caused primary osteoblasts to transform into osteosarcoma cells (135). Another study suggested that MET receptor is aberrantly expressed in almost all the human osteosarcoma cells (136). Other studies also found that inhibiting the MET signaling pathway can increase apoptotic rate and suppress the migration, proliferation, and invasion of osteosarcoma cells (137, 138). There is no report about the treatment of osteosarcoma with a selective MET inhibitor. However, the multi-target MET inhibitor cabozantinib has a high efficiency in osteosarcoma (Table 1 and Figure 3) (20). All this evidence suggests that MET may be one of the drivers of osteosarcoma.

### IGF-1R

IGF-1R is a hetero-tetrameric transmembrane glycoprotein with RTK activity, which is ubiquitously expressed in various human cell types and tissues (139). As an RTK, IGF-1R has a typical extracellular domain, transmembrane domain, and intracellular tyrosine kinase domain (136, 140). It plays an important role in growth and various physiological functions, including differentiation, development, apoptosis, and metabolism by binding to its ligand IGF-1 (141). Aberrant activation of IGF-1 has been observed in many malignancies (142). There are many new drugs being developed to target IGF/IGF-1R signaling pathway (139).

Regarding osteosarcoma, a study showed that the relative expression of IGF-1R in osteosarcoma was significantly higher than that in corresponding non-cancerous bones. The expression of IGF-1R is closely related to distant metastasis and prognosis of osteosarcoma. Higher IGF-1R expression in osteosarcoma patients is associated with poorer survival (143). Extensive screening and validation strategies identified IGF-1R as one of the specific RTKs that can activated and promote the phenotype of osteosarcoma cells *in vitro* (98). However, despite encouraging preclinical data, clinical trials of IGF-1R inhibitors for osteosarcoma have not yielded satisfactory results. In a phase II trial of the IGF-1R monoclonal antibody R1507 in patients with osteosarcoma, only two PRs were observed in 38 patients, with median PFS of 5.7 weeks (144). In another phase II trial of the IGF-1R antibody cixutumumab in children with osteosarcoma, no PRs were observed in 11 patients (Figure 3) (145). In general, IGF-1R might be one of the drivers of osteosarcoma, but inhibiting it alone is clearly not an option.

### AXL

AXL kinase contains a characteristically extracellular domain, transmembrane domain, and intracellular domain (146). Upon high-affinity binding to its ligand, growth arrest-specific protein 6 (GAS6), undergoes homodimerization, and subsequent transautophosphorylation within the intracellular kinase domain, thus activating downstream signaling pathways (147). The Gas6/AXL signaling pathway play an important role in many physiological processes (148). The aberrant expression of Gas6/AXL has been observed in many malignancies (147). Different small-molecule inhibitors and monoclonal antibodies of AXL have been developed (148).

There have been identified AXL as one of the specific RTKs that can activated and promote the phenotype of osteosarcoma cells *in vitro* (149). Higher AXL expression in osteosarcoma patients is associated with poorer survival (150). Knockdown of AXL in osteosarcoma cells leads to decreased proliferation and increased apoptosis (151). In addition, miR-199a-3p and lncRNA DANCR regulate the progression of osteosarcoma through targeting AXL (152). A retrospective study showed that one in five patients with osteosarcoma treated with an AXL inhibitor, sunitinib, showed PR (36). In an phase II trial of the AXL inhibitor cabozantinib in osteosarcoma, the 6-months PFS rate was 33%, the median PFS was 6.2 months, and the PR rate was 12% (Figure 3) (20). It should be noted that both sunitinib and cabozantinib are multi-target TKIs. At present, there is no report about the efficacy of single-target drugs against AXL in osteosarcoma. From the above evidence, we suspect that AXL is also a driver of osteosarcoma.

## Discussion

In this review, we first summarized the multitarget TKIs that were reported in registered clinical trials in patients with osteosarcoma. We compared the targets of these TKIs and found that VEGFRs and RET may be the key RTKs for the treatment of osteosarcoma. We further showed that KIT, PDGFRs, and FGFRs might be relevant but unimportant RTKs for osteosarcoma. In addition, we reviewed the literature and found that MET, IGF-1R, and AXL may also be relevant targets for the treatment of osteosarcoma.

Preclinical studies indicate that all eight RTKs mentioned above play an important role in the progression of osteosarcoma. However, clinical trials in osteosarcoma have demonstrated the low efficacy of single-target therapy by inhibiting VEGFs (by bevacizumab) (80), KIT and PDGFRα (by imatinib) (115), and IGF-1R (by cixutumumab or R1507) (Figure 3) (144, 145). The results of these clinical trials indicate that the inhibition of one type of RTKs in the treatment of osteosarcoma is not feasible. The molecular mechanism indicates that there is a large amount of crossover and overlap among the downstream signaling pathways of RTKs. For example, SRC kinase, which plays a key role in the development of osteosarcoma (153), can be activated, respectively by VEGFRs (154), KIT (81), RET (90), PDGFRs (103), IGF-1R (155), and AXL (148). Therefore, it can be determined that inhibition of one type of RTKs is not effective in the treatment of osteosarcoma. It is necessary to inhibit several key RTKs simultaneously in order to achieve a breakthrough in the treatment of osteosarcoma. For example, cabozantinib, which can simultaneously inhibit VEGFRs, KIT, RET, PDGFRβ, MET, and AXL (21, 22), has the best effect in the treatment of osteosarcoma, with a median PFS of 6.2 months, compared to other TKIs listed in Table 1 and Figure 3 (20).

This review serves as a good reference in the field of RTK-targeted osteosarcoma treatment. First, this study can provide reference for new drug research and development. For example, researchers have discontinued studies on several kinds of IGF-1R antibodies because of poor clinical trial results. However, these single-target TKIs are ideal for combination therapy. Researchers should try to use these single-target drugs in combination with other drugs. Secondly, it provides reference for drug screening. Researchers can roughly determine whether a drug is effective for osteosarcoma based on the literature review of the eight RTKs mentioned above. Finally, according to this review, we can treat osteosarcoma precisely to reduce the side effects and improve the curative effect. For example, the combination of the single-target drugs of abovementioned eight RTKs can not only maximize the efficacy but also avoid the side effects associated with ineffective targets.

Obviously, there is still a lot of important works need to be done. Firstly, there are errors in IC_50_ values of some targets of each drug (21, 22, 32). These errors will mislead researchers while selecting drugs and cause confusion during follow-up research. Therefore, it is necessary to correct the IC_50_ values of each target of each TKI. Secondly, controlled clinical trials are needed to compare the effectiveness of various TKIs in the treatment of osteosarcoma. There is little work in this area. The current clinical trials of TKIs in osteosarcoma are all about one drug. Only controlled clinical trials can accurately compare the efficiency differences of each TKIs in osteosarcoma. Finally, this study is only a preliminary summary of these RTKs. Relevant signaling pathways and mechanisms still need to be studied further.

We can say that the current use of multi-target TKIs in the treatment of osteosarcoma is only a stopgap. The main drawback of this stopgap approach is the side effects associated with ineffective targets. With the emergence of various selective TKIs, the combination therapy using different selective (or single-target) TKIs in osteosarcoma will be the future trend.

## Conclusion

Currently, TKIs with promising therapeutic effect for osteosarcoma include apatinib, cabozantinib, lenvatinib, regorafenib, and sorafenib. Key RTKs for osteosarcoma treatment may include VEGFRs and RET. The receptors MET, IGF-1R, AXL, PDGFRs, KIT, and FGFRs might be relevant but unimportant RTKs for osteosarcoma. Inhibition of one type of RTK is not effective in the treatment of osteosarcoma. It is necessary to inhibit several relevant RTKs simultaneously in order to achieve a breakthrough in osteosarcoma treatment.

## Author Contributions

ZT and WY: conceptualization. XN: methodology. ZT: software, writing, and original draft preparation. WY: resources. XN and WY: writing, review, and editing. All authors have read and agreed to the published version of the manuscript.

## Conflict of Interest

The authors declare that the research was conducted in the absence of any commercial or financial relationships that could be construed as a potential conflict of interest.
